# Effect of levothyroxine on major adverse cardiovascular events in patients with hypothyroidism and cardiovascular disease

**DOI:** 10.3389/fendo.2025.1640086

**Published:** 2025-08-26

**Authors:** Lijia Liu, Haiqing Zhang, Xiaowei Chen, Zuoxiang Liu, Houyu Zhao, Shizhan Ma, Meng Zhao, Peng Shen, Yexiang Sun, Hongbo Lin, Siyan Zhan, Jiajun Zhao, Feng Sun

**Affiliations:** ^1^ Department of Epidemiology and Biostatistics, School of Public Health, Peking University, Beijing, China; ^2^ Key Laboratory of Major Disease Epidemiology, Ministry of Education (Peking University), Beijing, China; ^3^ Department of Endocrinology, Shandong Provincial Hospital Affiliated to Shandong First Medical University, Jinan, Shandong, China; ^4^ Shandong Clinical Medical Centre of Endocrinology and Metabolism, Jinan, Shandong, China; ^5^ Shandong Institute of Endocrine and Metabolic Disease, Jinan, Shandong, China; ^6^ Research Center of Clinical Epidemiology, Peking University Third Hospital, Beijing, China; ^7^ Yinzhou District Center for Disease Control and Prevention of Ningbo, Ningbo, China

**Keywords:** levothyroxine, MACE, hypothyroidism, pharmacoepidemiology, cohort study

## Abstract

**Objective:**

This study aimed to evaluate the effectiveness of levothyroxine, compared to non-levothyroxine treatment, in preventing adverse cardiovascular events and mortality in patients with hypothyroidism and cardiovascular disease (CVD).

**Methods:**

This is a retrospective study utilizing medical record data from the Yinzhou Regional Health Care Database. The analysis included patients newly diagnosed with hypothyroidism between July 2006 and December 2021 who also had pre-existing CVD at the time when they received the first hypothyroidism diagnosis. The primary outcome measure was the occurrence of three-point major adverse cardiovascular events (3P-MACE), which included cardiovascular death, non-fatal myocardial infarction, and non-fatal stroke. Secondary outcomes comprised all-cause mortality, all-cause hospitalization, and cardiovascular-related hospitalization. Propensity score matching was used to match levothyroxine users with non-users on a 1:1 basis. Cox proportional hazard models were employed to compare the risk of outcomes between users and non-users, with hazard ratios (HRs) and 95% confidence intervals (CIs) reported.

**Results:**

In the matched cohort (*n* = 1,332 in each group), 417 patients experienced 3P-MACE. Compared to those not treated with levothyroxine, patients receiving levothyroxine showed a significantly reduced risk of 3P-MACE (HR, 0.67; 95% CI, 0.55–0.82, *p* < 0.01), all-cause death (HR, 0.24; 95% CI, 0.16–0.35, *p* < 0.01), all-cause hospitalization (HR, 0.23; 95% CI, 0.21–0.26, *p* < 0.01), and cardiovascular-related hospitalization (HR, 0.69; 95% CI, 0.59–0.82, *p* < 0.01).

**Conclusions:**

Levothyroxine may improve major cardiovascular outcomes and decrease all-cause hospital admissions in patients with hypothyroidism and CVD.

## Introduction

Hypothyroidism is a prevalent pathological condition characterized by the thyroid gland’s inability to produce adequate thyroid hormones to fulfill the body’s metabolic needs (1). Hypothyroidism is primarily classified into overt hypothyroidism (OH) and subclinical hypothyroidism (SCH). The prevalence of OH and SCH in the general population ranges from 0.3% and 3.7% in the USA and from 0.2% and 5.3% in Europe, with variations depending on the specific definitions applied (2–7). A large cross-sectional study in China showed that the weighted prevalence of OH and SCH was 1.02% and 12.93%, respectively in adults (8). Untreated hypothyroidism can result in severe adverse health effects, including hypertension, dyslipidemia, infertility, cognitive impairments, and neuromuscular dysfunction, which can ultimately lead to death (1, 2). Additionally, population-based studies have also illustrated that SCH increases the incidence of heart failure, coronary heart disease, and stroke and thereby increases the risk of cardiovascular mortality and all-cause mortality (9–12).

The standard treatment for hypothyroidism is thyroid hormone replacement therapy with oral levothyroxine (LT4) on an empty stomach (2, 13). The latest guidelines from major endocrine societies unanimously recommend levothyroxine monotherapy as the first-line treatment for hypothyroidism (14). Thyroid hormones exert significant effects on the cardiovascular system, profoundly influencing cardiac function (15). Some studies have shown that levothyroxine may benefit the patients with concomitant cardiovascular disease (CVD) (16–18).

Although previous studies have indicated an association between hypothyroidism and several CVD risk factors, the findings were not consistent, as some observed improved outcomes, whereas others failed to identify a significant benefit (19–21). Moreover, there is a lack of large-scale observational studies that provide evidence on the association between levothyroxine treatment and CVD outcomes in a Chinese population. Therefore, this retrospective cohort study aimed to determine whether levothyroxine can prevent CVD in Chinese patients with hypothyroidism. In addition to CVD, this study also focused on other secondary outcomes, including all-cause mortality, first hospital admission, and first cardiovascular-related hospitalization.

## Materials and methods

### Data source

We used data from the Yinzhou Regional Health Care Database (YRHCD). The YRHCD amalgamated longitudinal data from a variety of sources, including electronic medical records (EMRs), disease monitoring and management systems, death registries, and other healthcare services within the Yinzhou District of Ningbo City, China (22–25). The YRHCD encompasses a diverse array of data types: (1) demographic details derived from population census records; (2) prescription information, which includes brand and generic names, Anatomical Therapeutic Chemical (ATC) Classification of Medications codes, prescription dates, dispensed quantities, and textual usage instructions; (3) data on physical activity, lifestyle, and clinical measurements sourced from health check records and disease surveillance and management systems; (4) diagnostic details from EMRs, covering diagnosis names, types, codes (the International Classification of Diseases, Tenth Revision), and dates; (5) laboratory test results and imaging examination data; and (6) mortality information from the death registry.

### Participants and cohort

The study cohort comprised patients who received their first diagnosis of hypothyroidism and were diagnosed with CVD before their first hypothyroidism diagnosis between 1 July 2006 and 31 December 2021. To ensure newly diagnosed cases, we included only those with no prior diagnosis of hypothyroidism for at least 180 days before the initial diagnosis date. The index date was defined as the date of the first levothyroxine prescription following the first hypothyroidism diagnosis. For the non-user group, the index date was matched to the date of the first hypothyroidism diagnosis. The baseline period was established as the 180 days preceding the index date, inclusive of the index date. During this period, patient data were collected to establish baseline characteristics and covariates.

### Outcome and follow-up

The primary outcome in this study is the incidence of the first three-point major adverse cardiovascular event (3P-MACE), defined as the occurrence of cardiovascular mortality, non-fatal myocardial infarction, or non-fatal stroke. The secondary outcomes assessed in this study were all-cause mortality, hospitalization, and CVD-related hospitalization. All-cause mortality was identified through death registry records. Hospitalization data included the date of admission and the reason for admission, which were coded using ICD classifications. Patients were then followed from the index date until the first occurrence of the following events: an outcome event, death (for outcomes other than mortality), loss to follow-up, or the study end date of 31 December 2022, whichever occurred first.

### Statistical analyses

Descriptive statistics were utilized to summarize baseline covariates. To mitigate potential confounding bias and achieve balance in baseline covariates, a propensity score matching (PSM)-based methodology was used for primary and secondary outcome analyses. The PS was estimated via a multivariable logistic regression model, with levothyroxine use as the dependent variable and baseline covariates as predictors. Following PS estimation, each patient in the levothyroxine user group was matched to a patient in the non-user group using the nearest-neighbor method. The balance of covariates was assessed using standardized differences, with an absolute value <0.1 indicating a balance. Some variables’ SMD remained above 0.1 after PSM, and they were adjusted in the subsequent Cox regression models. In the PS-matched population, a Cox regression model was used to estimate the hazard ratio (HR) with a 95% confidence interval (CI), assessing the outcomes between levothyroxine users and non-users. The proportional hazards assumption was tested using scaled Schoenfeld residuals. Time-to-event analyses were conducted using the Kaplan–Meier method, with appropriate graphical summaries.

Statistical analysis was performed using R software version 4.2.2. The R package “mice” was used for missing data imputation. All statistical tests were conducted two-sided and a *p*-value of less than 0.05 was considered to indicate statistical significance. The study was approved by the ethics committee of Peking University Health Science Center (IRB00001052-23157) and Shandong Provincial Hospital (SWYX: No. 2023-557). Informed consent was not required owing to the use of anonymized routine data.

### Subgroup analysis

A subgroup analysis based on baseline characteristics was conducted with particular interest in comparing the effectiveness of levothyroxine among patients with different age stages, sex, smoking status, drinking status, and type of hypothyroidism. All analyses were repeated separately for these subgroups in both the primary and secondary outcome analyses.

### Sensitivity analysis

Several sensitivity analyses were conducted to evaluate the robustness of the study findings. First, an analysis was performed without applying PSM to examine the effects of levothyroxine use on the outcomes by multivariate Cox regression models. Second, in analyzing the primary and secondary objectives, we incorporated the Charlson Comorbidity Index (CCI) along with relevant baseline variables to adjust for comorbidities (26). The third sensitivity analysis focused on patients who had not experienced MACE within 6 months before their first hypothyroidism diagnosis. In the fourth sensitivity analysis, we limited the study to subjects whose laboratory markers [thyroid-stimulating hormone (TSH), FT3, and FT4] met the criteria for the initial diagnosis of hypothyroidism (SCH: TSH > 4.8 mIU/L, FT3 9–25 pmol/L and FT4 2.1–5.4 pmol/L; OH: TSH > 4.8 mIU/L and FT4 < 9 pmol/L), ensuring consistency in the diagnostic definition. Fifth, the Fine-Gray subdistribution hazard model was used to check possible competing risks by all-cause death when analyzing MACE, all-cause hospitalization, and CVD-related hospitalization. Sixth, we applied alternative PSM methods, caliper matching with a caliper value of 0.2, to assess their impact on the results. Seventh, we excluded patients in the user group who did not have their first prescription at the time of the first hypothyroidism diagnosis to exclude immortal time. Eighth, patients who had incident outcomes within 30 days (latent period) after the index date were excluded, to control reverse causation and unmeasured confounding by undiagnosed disease (27). Finally, we broadened the exposure definition by classifying any patient with at least one levothyroxine prescription record as treated.

## Results

### Basic characteristics

**Figure 1:**
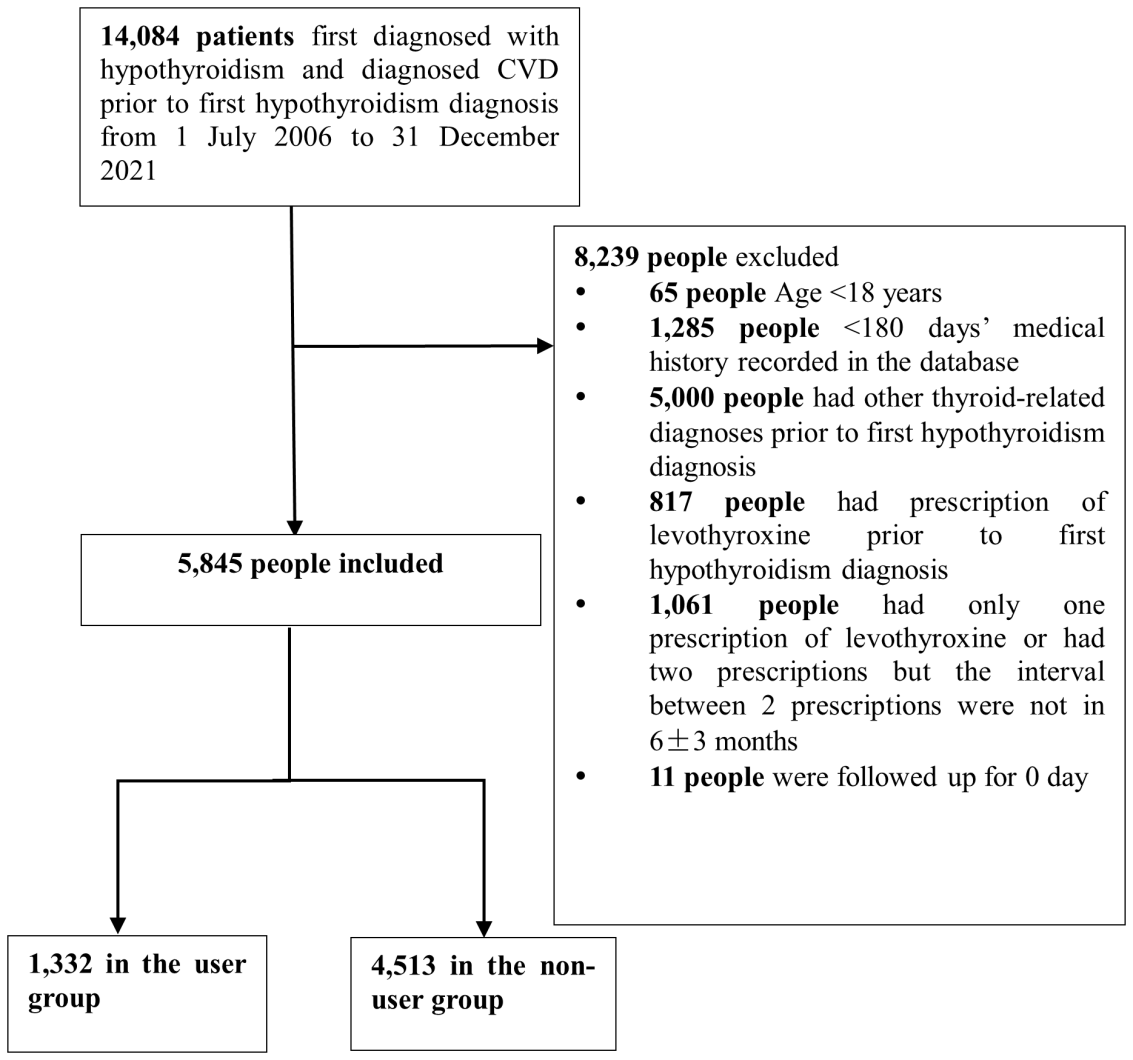
Flowchart of participants in the study of primary outcome (3P-MACE).

The primary cohort ultimately included 5,845 patients (age 65.2 ± 15.3 years, 60.4% female), of whom 1,332 (22.8%) were treated with levothyroxine (**Figure 1**). Before PS matching, compared with non-users, the medication group had a higher proportion of people over 45 years of age, a higher proportion of the urban population, a higher proportion of smokers and alcohol drinkers, and a lower proportion of hypertension, CVD, and diabetes, and the proportion of those who received cardiovascular system medications, antithrombotic agents, anti-diabetic medications, and nervous system medications was lower (**Table 1**). Baseline covariates for inclusion into the PS model included sex (male or female), age at index date, smoking status, alcohol status, body mass index (BMI) categories, hypertension, hyperlipemia, diabetes mellitus, cardiovascular system medication, antithrombotic agent, anti-diabetic medication, nervous system medication, FT3 level, FT4 level, and TSH level. After PSM, covariates except hyperlipemia and FT4 were effectively balanced between the exposure and comparison groups.

**Table 1:** Characteristics of included participants 3P-MACE (before and after PSM).

Characteristics	Pre-PS matching			Post-PS matching		
	Non-user group (N=4513)	User group (N=1332)	SMD	Non-user group (n=1,332)	User group (n=1,332)	SMD
Sex, N(%)			0.064			0.023
Male	2695 (59.7%)	837 (62.8%)		852 (64.0%)	837 (62.8%)	
Female	1818 (40.3%)	495 (37.2%)		480 (36.0%)	495 (37.2%)	
Residence, N(%)			0.431			0.411
Urban	2575 (57.1%)	480 (36.0%)		747 (56.1%)	480 (36.0%)	
Rural	1938 (42.9%)	852 (64.0%)		585 (43.9%)	852 (64.0%)	
Health insurance, N(%)			0.289			0.315
None	688 (15.2%)	110 (8.3%)		224 (16.8%)	110 (8.3%)	
Others	208 (4.6%)	22 (1.7%)		56 (4.2%)	22 (1.7%)	
Private	1 (0.0%)	1 (0.1%)		0 (0%)	1 (0.1%)	
Public	3616 (80.1%)	1199 (90.0%)		1052 (79.0%)	1199 (90.0%)	
Age at index date, N(%)			0.276			0.015
18-59 years	1306 (28.9%)	560 (42.0%)		570 (42.8%)	560 (42.0%)	
≥60 years	3207 (71.1%)	772 (58.0%)		762 (57.2%)	772 (58.0%)	
BMI, N(%)			0.087			0.008
<18.5	230 (5.1%)	50 (3.8%)		51 (3.8%)	50 (3.8%)	
18.5-28	3692 (81.8%)	1130 (84.8%)		1126 (84.5%)	1130 (84.8%)	
≥28	591 (13.1%)	152 (11.4%)		155 (11.6%)	152 (11.4%)	
Smoking status, N(%)			0.196			0.031
No	3536 (78.4%)	930 (69.8%)		949 (71.2%)	930 (69.8%)	
Yes	977 (21.6%)	402 (30.2%)		383 (28.8%)	402 (30.2%)	
Alcohol status, N(%)			0.1			0.059
No	3903 (86.5%)	1104 (82.9%)		1133 (85.1%)	1104 (82.9%)	
Yes	610 (13.5%)	228 (17.1%)		199 (14.9%)	228 (17.1%)	
FT3, pmol/L			0.043			0.035
Median (Q1,Q3)	4.31 (3.80,4.78)	4.40 (3.89,4.81)		4.40 (3.89,4.81)	4.40 (3.89,4.81)	
FT4, pmol/L			0.472			0.225
Median (Q1,Q3)	12.6 (10.4,15.2)	10.7 (8.35,13.5)		11.4 (9.55,13.4)	10.7 (8.35,13.5)	
TSH, mU/L			0.016			0.007
Median (Q1,Q3)	5.92 (5.14,7.75)	7.22 (5.38,15.3)		5.94 (5.15,7.91)	7.22 (5.38,15.3)	
Hypertension, N(%)			0.224			0.006
No	2189 (48.5%)	794 (59.6%)		798 (59.9%)	794 (59.6%)	
Yes	2324 (51.5%)	538 (40.4%)		534 (40.1%)	538 (40.4%)	
Hyperlipidemia, N(%)			0.112			0.113
No	4095 (90.7%)	1162 (87.2%)		1209 (90.8%)	1162 (87.2%)	
Yes	418 (9.3%)	170 (12.8%)		123 (9.2%)	170 (12.8%)	
Diabetes mellitus, N(%)			0.213			0.004
No	4151 (92.0%)	1290 (96.8%)		1289 (96.8%)	1290 (96.8%)	
Yes	362 (8.0%)	42 (3.2%)		43 (3.2%)	42 (3.2%)	
Cardiovascular system medication, N(%)			1.283			0.039
No	2289 (50.7%)	1304 (97.9%)		1311 (98.4%)	1304 (97.9%)	
Yes	2224 (49.3%)	28 (2.1%)		21 (1.6%)	28 (2.1%)	
Anti-diabetic medication, N(%)			0.256			0.012
No	4318 (95.7%)	1326 (99.5%)		1327 (99.6%)	1326 (99.5%)	
Yes	195 (4.3%)	6 (0.5%)		5 (0.4%)	6 (0.5%)	
Antithrombotic agents, N(%)			0.415			0.038
No	3865 (85.6%)	1293 (97.1%)		1284 (96.4%)	1293 (97.1%)	
Yes	648 (14.4%)	39 (2.9%)		48 (3.6%)	39 (2.9%)	
Nervous system medication, N(%)			0.372			0.027
No	4068 (90.1%)	1313 (98.6%)		1317 (98.9%)	1313 (98.6%)	
Yes	445 (9.9%)	19 (1.4%)		15 (1.1%)	19 (1.4%)	

*Baseline covariates for inclusion into the PS model will include: sex (male, female), age at index date, smoking status, alcohol status, BMI categories, hypertension, hyperlipemia, diabetes mellitus, cardiovascular system medication, antithrombotic agent, anti-diabetic medication, nervous system medication, FT3 level, FT4 level, and TSH level

*SMD, standardized mean difference (absolute SMD ≥ 0.10 indicates covariate imbalance)

The secondary outcome cohort ultimately included 5,843 patients, comprising 1,332 levothyroxine users and 4,511 non-users (**Supplementary Figure 1**). The baseline characteristics before and after PS matching are detailed in **Supplementary Tables 1–3**.

### Primary analyses

#### Primary outcome 3P-MACE

**Figure 2:**
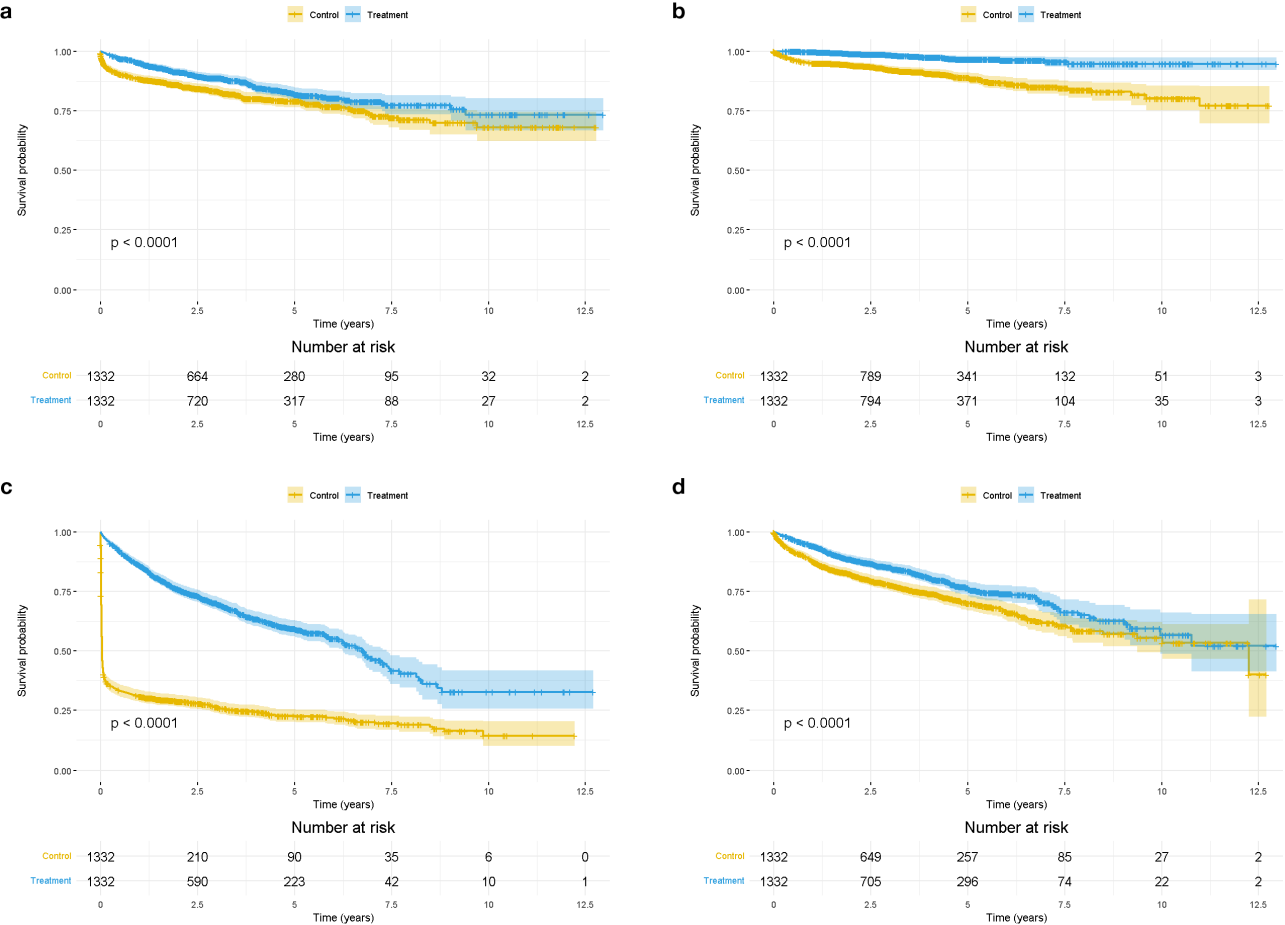
Kaplan-Meier survival curve of primary and secondary outcomes by levothyroxine use **(a)**. The Kaplan-Meier survival curve of 3P-MACE by treatment group **(b)**. The Kaplan-Meier survival curve of all-cause death by treatment group **(c)**. The Kaplan-Meier survival curve of all-cause hospitalization by treatment group **(d)**. The Kaplan-Meier survival curve of cardiovascular-related hospitalization by treatment group. *P for interaction <0.05.

In the primary analyses, during a median follow-up time of 3.22 (95% CI, 3.11–3.38) years, 1,285 new-onset 3P-MACE occurred. The incidences of 3P-MACE were 400.17 and 327.17 per 1,000 person-years in non-users and levothyroxine users, respectively (**Table 2**). After PS matching, there were 417 cases of new-onset 3P-MACE. The Kaplan–Meier survival curve of 3P-MACE by treatment group is presented in **Figure 2a**. Levothyroxine use was associated with a lower risk of 3P-MACE incidence, with an HR of 0.67 (95% CI, 0.55–0.82) in the Cox model (*p* < 0.01).

**Table 2:** Association between treatment and primary and secondary outcomes.

Outcome	Levothyroxine use	Before PS matching		After PS matching		HR (95% CI)	P value
		Events	Rate per 1000 person-years	Events	Rate per 1000 person-years		
3P-MACE	Non-user	1108	400.17	240	374.69	1.00	< 0.01
	User	177	327.17	177	327.17	0.67 (0.55, 0.82)	
All-cause death	Non-user	727	302.20	125	296.50	1.00	< 0.01
	User	29	270.69	29	270.69	0.24 (0.16, 0.35)	
All-cause hospitalization	Non-user	3570	1809.52	991	1582.62	1.00	< 0.01
	User	445	472.97	445	472.97	0.23 (0.21, 0.26)	
CVD related hospitalization	Non-user	1649	484.95	326	405.75	1.00	< 0.01
	User	244	354.34	244	354.34	0.69 (0.59, 0.82)	

*The outcome of 3P-MACE, all-cause death, CVD related hospitalization adjusted FT4 and Hyperlipidemia in the Cox model; The outcome of all-cause hospitalization adjusted FT4 in the Cox model

*Two-sided P values comparing the treatment and non-treatment groups

#### Secondary outcome

Before PS matching, in the secondary outcome cohort, during a median follow-up time of 3.64 years (95% CI, 3.57–3.71), there were 756 incidents of all-cause death. The incidence rates of all-cause death were 302.20 and 270.69 per 1,000 person-years in non-users and users, respectively (**Table 2**). **Figure 2b** shows the Kaplan–Meier survival curve of all-cause death by treatment group after PS matching. A total of 154 new-onset all-cause deaths occurred. Levothyroxine use was associated with a reduced risk of all-cause death, with an HR of 0.24 (95% CI, 0.16–0.35).

The study also examined all-cause hospitalization. Before PS matching, there were a total of 4,015 new all-cause hospitalization. After matching, the number was 1,436 (**Table 2**). **Figure 2c** displays the Kaplan–Meier survival curve of all-cause hospitalization by treatment group. Levothyroxine use was associated with a significantly lower risk of all-cause hospitalization, with an HR of 0.23 (95% CI, 0.21–0.26).

During a median follow-up time of 3.41 (95% CI, 3.28–3.54) years, 1,893 incident cardiovascular-related hospitalization occurred. The incidences were 484.95 per 1,000 person-years in non-users and 354.34 per 1,000 person-years in users (**Table 2**). After PS matching, 570 new-onset cardiovascular-related hospitalization occurred. **Figure 2d** shows the Kaplan–Meier survival curve of cardiovascular-related hospitalization by treatment group. Levothyroxine use reduced the risk of CVD-related hospitalization, with an HR of 0.69 (95% CI, 0.59–0.82).

### Subgroup analyses

**Figure 3:**
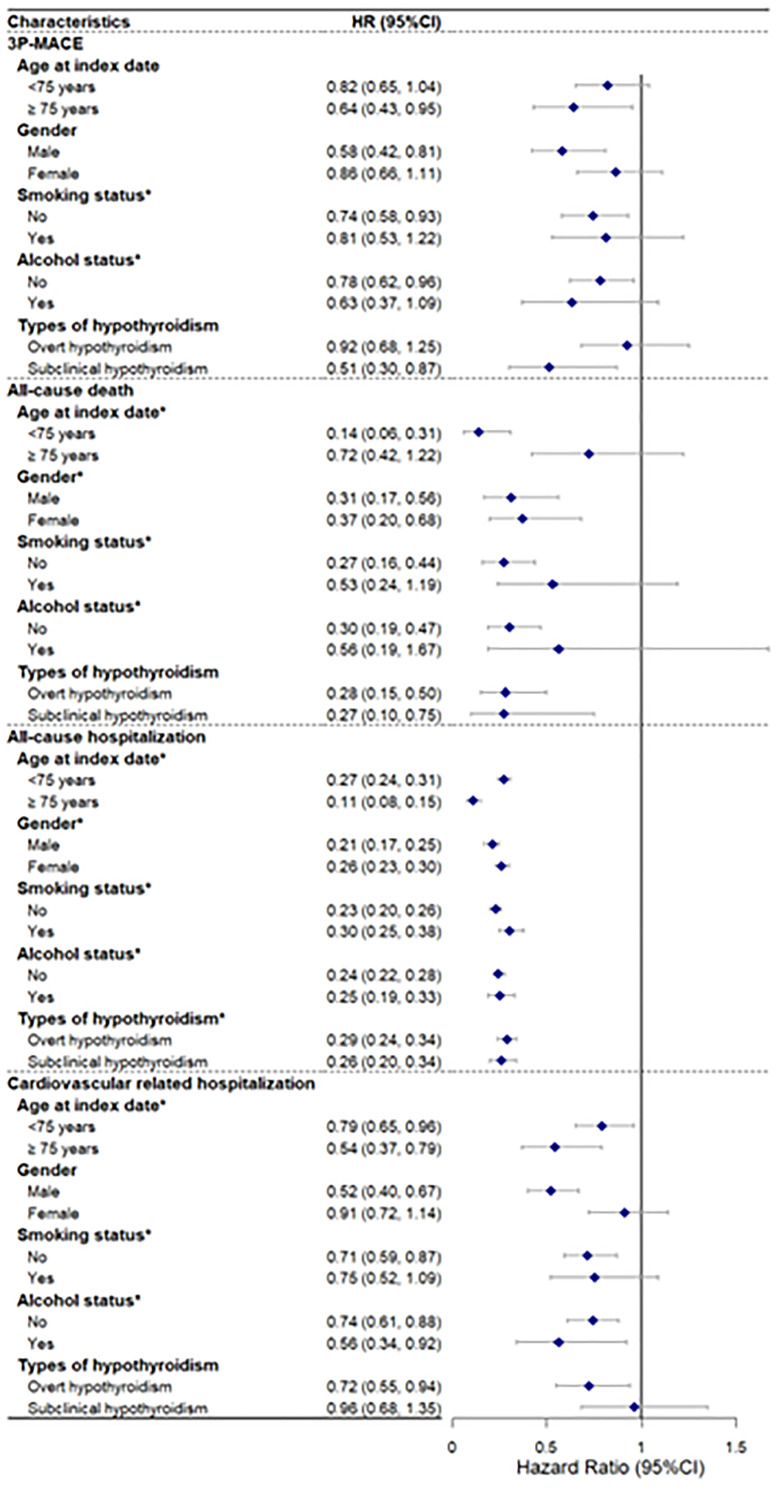
Subgroup analysis of association between treatment and primary and secondary outcomes.

In the subgroup analysis, the results showed consistent associations across different subgroups (**Figure 3**). Notably, smoking status and alcohol status had significant interactions for all outcomes, and age and gender had significant interactions for all-cause death and all-cause hospitalization, suggesting that these factors may modify the effect of the drug on these outcomes. Moreover, this study did not find differences between OH and SCH in the protective effects of levothyroxine on 3P-MACE, all-cause death, and CVD-related hospitalization. More details can be found in **Supplementary Table 4**.

### Sensitivity analyses

The results of sensitivity analyses are summarized in **Table 3** (**Supplementary Figure 2**). First, when the analysis was performed without PSM, levothyroxine use was still associated with a lower risk of 3P-MACE, with an HR of 0.64 (95% CI, 0.54–0.77). The risks of all-cause death, all-cause hospitalization, and cardiovascular-related hospitalization were also reduced. Second, after including baseline CCI in PS matching, all Cox regression models showed consistent results with the primary analysis. Third, we excluded patients who had MACE within 6 months prior to their first diagnosis; the HR for 3P-MACE was 0.74 (95% CI, 0.60–0.90). The reduction in the risk of all-cause death, all-cause hospitalization, and CVD-related hospitalization also remained significant. Fourth, when the analysis was restricted to patients whose laboratory markers (TSH, FT3, and FT4) fulfilled the first diagnosis of hypothyroidism, the model for 3P-MACE showed no significant reduction (HR, 0.79; 95% CI, 0.55–1.14), while the risks of all-cause death, all-cause hospitalization, and CVD-related hospitalization were significantly lower. Fifth, the competing risk models presented that the results of HR were in line with that of the primary analysis, indicating no significant competing risk due to all-cause mortality. Sixth, after applying matching with a caliper value of 0.2, we obtained consistent HR estimations. Seventh, we excluded patients in the user group who did not have their first prescription at the time of the first hypothyroidism diagnosis, and the Cox models showed that the direction of HR was inconsistent with that of the primary analysis though there was no statistical difference for 3P-MACE. Eighth, after excluding patients who had outcome events within 30 days after the index date, the Cox models displayed a similar direction of HR. Finally, when levothyroxine exposure was broadened to include patients with at least one prescription, the treatment group rose from 1,332 to 2,383 individuals. Applying the same analytic approach as in the primary analysis, levothyroxine therapy remained significantly associated with reduced risks of 3P-MACE, all−cause mortality, all−cause hospitalization, and cardiovascular-related hospitalization. The sensitivity analyses confirmed the robustness of the primary and secondary outcome results.

**Table 3:** Sensitivity analysis of association between treatment and primary and secondary outcomes.

Sensitivity analysis	Outcomes [HR (95%CI)] *			
	3P-MACE	All-cause death	All-cause hospitalization	CVD related hospitalization
NO PSM	0.64 (0.54, 0.77)	0.22 (0.15, 0.33)	0.22 (0.20, 0.25)	0.62 (0.54, 0.73)
Combined CCI	0.71 (0.58, 0.86)	0.26 (0.17, 0.39)	0.23 (0.21, 0.26)	0.66 (0.56, 0.78)
Exclusion of 3pmace within 6 months prior to the first diagnosis	0.74 (0.60, 0.90)	0.22 (0.15, 0.33)	0.23 (0.21, 0.26)	0.65 (0.55, 0.77)
Restriction of study subjects whose laboratory markers (TSH, FT3, FT4) fulfilled the first diagnosis of hypothyroidism	0.79 (0.55, 1.14)	0.30 (0.14, 0.64)	0.23 (0.18, 0.28)	0.67 (0.49, 0.92)
Competitive risk model	0.77 (0.64,0.94)	—	0.23 (0.21, 0.26)	0.67 (0.57, 0.79)
PS matching using a caliper width of 0.2	0.67 (0.55, 0.82)	0.25 (0.17, 0.38)	0.24 (0.21, 0.27)	0.70 (0.59, 0.83)
Matching the index date	0.76 (0.58, 1.01)	0.24 (0.14, 0.41)	0.21 (0.18, 0.25)	0.59 (0.46, 0.76)
Exclusion of new onset outcomes within 30 days after the index date	0.89 (0.72, 1.10)	0.27 (0.18, 0.40)	0.76 (0.66, 0.89)	0.72 (0.60, 0.85)
Including individuals with only single medication record in the treatment group	0.72 (0.62, 0.83)	0.28 (0.22, 0.35)	0.24 (0.22, 0.26)	0.60 (0.53, 0.68)

*If the SMD between some variables remained above 0.1 after PS matching, they were further adjusted in the Cox models.

## Discussion

This retrospective cohort study found that levothyroxine use was associated with a significant reduction in the risk of 3P-MACE, all-cause death, all-cause hospitalization, and cardiovascular-related hospitalization. Primary results remained consistent in subgroup analyses and sensitivity analyses.

Levothyroxine is a commonly used therapeutic agent for hypothyroidism and may be beneficial to reduce CVD risk. Patients with hypothyroidism have elevated serum TSH and consecutively elevated levels of total cholesterol and low-density lipoprotein cholesterol (LDL-C) (28). Levothyroxine can promote the excretion of neutral cholesterol in feces (cholesterol and its derivatives such as bile acids), improve lipoprotein metabolism and anti-inflammatory and anti-oxidative stress, help reduce LDL-C and HDL-C levels, and thereby may improve lipid profiles, reduce vascular resistance, and enhance endothelial function, contractility, and cardiac mitochondrial function, which, in turn, improves cardiac function (29–32). Recent studies have further clarified the roles of thyroid hormones in cardiac remodeling and function, especially following coronary artery disease. Thyroid hormone signaling pathways, mediated by thyroid hormone receptors, can enhance myocardial mitochondrial function, reduce cardiac fibrosis, and improve endothelial function, thereby mitigating pathological cardiac remodeling and dysfunction following ischemic injury (33–35). Specifically, evidence from clinical studies indicates that acute triiodothyronine administration after myocardial infarction may improve ventricular remodeling and cardiac performance, potentially by promoting favorable myocardial metabolic adaptations and exerting anti-inflammatory effects (34). Additionally, preclinical research supports thyroid hormones’ beneficial effects on cardiomyocyte regeneration and repair mechanisms, further underscoring the potential for thyroid hormone-based treatments to positively impact long-term cardiovascular outcomes (35). These insights provide mechanistic context for the cardiovascular benefits associated with levothyroxine therapy observed in our study. When patients with SCH were treated with levothyroxine replacement therapy, in addition to the resolution of hypothyroidism symptoms, these patients also had improvement in atherogenic lipoprotein profiles, arterial stiffness, and intima-media thickening (30, 31, 36).

The available evidence on the protective effect of levothyroxine on adverse cardiovascular events in patients with hypothyroidism is mixed. For example, Razvi et al. analyzed the Whickham Survey and found that the treatment of SCH with levothyroxine appeared to attenuate ischemic heart disease (IHD)-related morbidity and mortality (16), but the sample size was small (SCH group *n* = 91). Another larger cohort study by Razvi et al. (18), based on the United Kingdom General Practitioner Research Database (CPRD), showed that levothyroxine treatment was associated with a reduction in IHD in younger individuals (aged 40–70 years), whereas this effect was not evident in older patients (aged >70 years). However, our study did not find a difference in the beneficial effects of levothyroxine therapy between younger and older patients. Compared to another study using the CPRD focused mainly on SCH, tightly defining the population by TSH ranges of 5–10 mU/L (37), we examined an OH and SCH population with established CVD and adjusted for key thyroid function parameters. Thereby, our study reflected a higher-risk population more representative of routine clinical practice. A systematic review and meta-analysis demonstrated that levothyroxine supplementation was effective in improving surrogate endpoints for cardiovascular events such as cardiac function indices, cardiac output, and left ventricular ejection fraction in patients with SCH (38). However, some studies have shown that, in some cases, levothyroxine treatment may not significantly decrease the risk of cardiovascular outcomes (19, 21, 39, 40). In a large real-world cohort study in Denmark, levothyroxine treatment in patients with SCH and heart disease was not associated with a significant benefit or risk of all-cause mortality, MACE, or hospital admission (40). Several randomized controlled trials also indicated that levothyroxine provided no apparent benefits (cardiovascular events, cardiovascular mortality, etc.) in older persons with SCH (21, 39). This suggests that the effects of levothyroxine therapy on CVD may vary according to individual differences (17). In addition, previous studies examining the association between levothyroxine use and cardiovascular outcomes have often neglected to consider thyroid status (19, 40), while in our study, TSH, FT3, and FT4 levels were adjusted in the matching analysis.

Notably, even after broadening our definition of levothyroxine treatment to include patients with at least one prescription during follow−up, the treatment rate among Chinese patients with hypothyroidism and CVD remained relatively low at 34.6 %. Our primary definition was designed to capture patients with sustained medication and a high probability of achieving normalization of thyroid function, because most studies indicate that a minimum of 4–6 months of continuous therapy is necessary for plasma TSH and FT4 levels to normalize (41). Nevertheless, treatment rates under both definitions were substantially lower than those reported in developed countries. For comparison, recent reports from Europe and the United States indicated significantly higher levothyroxine treatment rates, generally exceeding 60%–80% (42, 43).

To our knowledge, this is the first study to report levothyroxine treatment rates in Chinese patients with both hypothyroidism and CVD. The observed rate is similar to that of diabetes, another prevalent endocrine disorder in China, whose national treatment rate is approximately 32.4% (44). Although the underlying reasons for these low treatment rates are complex and multifactorial, contributing factors probably include environmental, socioeconomic, lifestyle, and health−policy influences specific to China (44–49). Our findings underscore the importance of appropriate levothyroxine therapy in patients with hypothyroidism, particularly those with co-existing CVD. Timely and adequate treatment of hypothyroidism can mitigate cardiovascular risks, improve patient outcomes, and potentially reduce healthcare costs associated with cardiovascular events and hospitalizations.

As far as we are aware, this study was the first cohort to assess the association between levothyroxine use and incident cardiovascular outcomes and hospital admissions in a Chinese population. Our study had several strengths, including the use of a real-world database, which provides evidence for post-marketing surveillance (PMS) for drugs. We adjusted for potential confounding factors through PSM and multivariable analysis, mitigating confounding by indication (50). In addition, our study specifically investigates the cardiovascular protective effects of levothyroxine treatment in patients with hypothyroidism with pre-existing CVD, which may provide additional insights beyond previous research. Moreover, we performed multiple subgroup analyses and sensitivity analyses, which confirmed the robustness of our results.

However, there were several limitations to our study. First, the patients enrolled in the study were from a single municipal district in China, which may limit the external validity of the findings. Second, despite adjustment for various baseline confounding factors, important time-varying covariates were not considered and residual confounding cannot be entirely excluded. Third, in this study, the initial points of exposure for the two groups were not entirely consistent, which may lead to immortal-time bias and overestimate the protective effect of the medication (51). However, since approximately two-thirds of the users in the cohort were prescribed their first levothyroxine prescription at the time of their first hypothyroidism diagnosis, we set the index date for the non-user group as the date of their first hypothyroidism diagnosis. The control group was much larger than the exposure group in our study, and immortal time accounted for a smaller proportion of the follow-up time in the exposure group; thus, immortal-time bias induced was relatively small (52). Fourth, the database did not record precise dosage information of drugs, and we did not explore the dose–response effect between levothyroxine use and 3P-MACE risk. Finally, this study mainly used coded information and did not conduct case validation for patients with cardiovascular events, which may lead to misclassification bias of outcomes.

## Conclusion

In conclusion, this retrospective cohort study demonstrated that levothyroxine therapy was associated with a lower risk of major adverse cardiovascular events, all-cause mortality, and hospitalizations in Chinese patients with hypothyroidism. These results highlight the clinical benefits of levothyroxine and emphasize the importance of managing hypothyroidism to improve cardiovascular outcomes. Further prospective studies and randomized trials are needed to explore its long-term benefits and mechanisms.

## Data Availability

The raw data supporting the conclusions of this article will be made available by the authors, without undue reservation.
