# Supplementary material for: Effect of levothyroxine on major adverse cardiovascular events in patients with hypothyroidism and cardiovascular disease
Source: Front Endocrinol (Lausanne). 2025 Aug 26;16:1640086. doi: 10.3389/fendo.2025.1640086 (PMC12417138; doi:10.3389/fendo.2025.1640086)
Supplement: Supplementary file 1 [file DataSheet1.docx]

**Supplementary Figure S1. Flowchart of participants in the study of secondary outcomes (all-cause death, first hospital hospitalization, first CVD related** **hospitalization)**

**14,084 patients** first diagnosed with hypothyroidism and diagnosed CVD prior to first hypothyroidism diagnosis from 1 July 2006 to 31 December 2021

**8,241 people** excluded

- **65 people** Age <18 years
- **1,285 people** <180 days’ medical history recorded in the database
- **5,000 people** had other thyroid-related diagnoses prior to first hypothyroidism diagnosis
- **817 people** had prescription of levothyroxine prior to first hypothyroidism diagnosis
- **1,061 people** had only one prescription of levothyroxine or had two prescriptions but the interval between 2 prescriptions were not in 6±3 months
- **13 people** were followed up for 0 day

**5,843 people included**

**1,332 in the user group**

**4,511 in the non-user group**

**Supplementary Table S1. Characteristics of included participants all-cause death (before and after PSM)**

|  | Pre-PS matching | | |  | Post-PS matching | | |
| --- | --- | --- | --- | --- | --- | --- | --- |
| Characteristics | Non-user group (n=4,511) | User group (n=1,332) | SMD |  | Non-user group (n=1,332) | User group (n=1,332) | SMD |
| Sex, N(%) |  |  | 0.071 |  |  |  | 0.002 |
| Male | 2688 (59.6%) | 840 (63.1%) | |  | 839 (63.0%) | 840 (63.1%) | |
| Female | 1823 (40.4%) | 492 (36.9%) | |  | 493 (37.0%) | 492 (36.9%) | |
| Residence, N(%) | |  | 0.426 |  |  |  | 0.398 |
| Urban | 2587 (57.3%) | 487 (36.6%) | |  | 746 (56.0%) | 487 (36.6%) | |
| Rural | 1924 (42.7%) | 845 (63.4%) | |  | 586 (44.0%) | 845 (63.4%) | |
| Health insurance, N(%) | | | 0.284 |  |  |  | 0.323 |
| None | 690 (15.3%) | 115 (8.6%) | |  | 223 (16.7%) | 115 (8.6%) | |
| Others | 205 (4.5%) | 20 (1.5%) |  |  | 63 (4.7%) | 20 (1.5%) |  |
| Private | 1 (0.0%) | 1 (0.1%) |  |  | 0 (0%) | 1 (0.1%) |  |
| Public | 3615 (80.1%) | 1196 (89.8%) | |  | 1046 (78.5%) | 1196 (89.8%) | |
| Age at index date, N(%) | | | 0.277 |  |  |  | 0.024 |
| 18-60 years | 1305 (28.9%) | 560 (42.0%) | |  | 576 (43.2%) | 560 (42.0%) | |
| ≥60 years | 3206 (71.1%) | 772 (58.0%) | |  | 756 (56.8%) | 772 (58.0%) | |
| BMI, N(%) | |  | 0.099 |  |  |  | 0.026 |
| ＜18.5 | 299 (6.6%) | 59 (4.4%) |  |  | 66 (5.0%) | 59 (4.4%) |  |
| 18.5-28 | 3619 (80.2%) | 1104 (82.9%) | |  | 1094 (82.1%) | 1104 (82.9%) | |
| ≥28 | 593 (13.1%) | 169 (12.7%) | |  | 172 (12.9%) | 169 (12.7%) | |
| Smoking status, N(%) | |  | 0.226 |  |  |  | 0.033 |
| No | 3534 (78.3%) | 911 (68.4%) | |  | 931 (69.9%) | 911 (68.4%) | |
| Yes | 977 (21.7%) | 421 (31.6%) | |  | 401 (30.1%) | 421 (31.6%) | |
| Alcohol status, N(%) | |  | 0.117 |  |  |  | 0.043 |
| No | 3873 (85.9%) | 1086 (81.5%) | |  | 1108 (83.2%) | 1086 (81.5%) | |
| Yes | 638 (14.1%) | 246 (18.5%) | |  | 224 (16.8%) | 246 (18.5%) | |
| FT3, pmol/L | |  | 0.032 |  |  |  | 0.003 |
| Median (Q1,Q3) | 4.31 (3.80,4.79) | 4.37 (3.87,4.84) | |  | 4.38 (3.85,4.82) | 4.37 (3.87,4.84) | |
| FT4, pmol/L | |  | 0.428 |  |  |  | 0.177 |
| Median (Q1,Q3) | 12.6 (10.4,15.2) | 10.9 (8.62,13.8) | |  | 11.3 (9.51,13.4) | 10.9 (8.62,13.8) | |
| TSH, mU/L | |  | 0.019 |  |  |  | 0.03 |
| Median (Q1,Q3) | 5.90 (5.15,7.75) | 6.96 (5.38,15.6) | |  | 5.91 (5.17,7.76) | 6.96 (5.38,15.6) | |
| Hypertension, N(%) | |  | 0.224 |  |  |  | 0.012 |
| No | 2189 (48.5%) | 794 (59.6%) | |  | 802 (60.2%) | 794 (59.6%) | |
| Yes | 2322 (51.5%) | 538 (40.4%) | |  | 530 (39.8%) | 538 (40.4%) | |
| Hyperlipidemia, N(%) | |  | 0.112 |  |  |  | 0.108 |
| No | 4093 (90.7%) | 1162 (87.2%) | |  | 1207 (90.6%) | 1162 (87.2%) | |
| Yes | 418 (9.3%) | 170 (12.8%) | |  | 125 (9.4%) | 170 (12.8%) | |
| Diabetes mellitus, N(%) | | | 0.213 |  |  |  | 0.041 |
| No | 4149 (92.0%) | 1290 (96.8%) | |  | 1280 (96.1%) | 1290 (96.8%) | |
| Yes | 362 (8.0%) | 42 (3.2%) |  |  | 52 (3.9%) | 42 (3.2%) |  |
| Cardiovascular system medication, N(%) | | | 1.283 |  |  |  | 0.027 |
| No | 2288 (50.7%) | 1304 (97.9%) | |  | 1309 (98.3%) | 1304 (97.9%) | |
| Yes | 2223 (49.3%) | 28 (2.1%) |  |  | 23 (1.7%) | 28 (2.1%) |  |
| Anti-diabetic medication, N(%) | | | 0.256 |  |  |  | 0.011 |
| No | 4316 (95.7%) | 1326 (99.5%) | |  | 1325 (99.5%) | 1326 (99.5%) | |
| Yes | 195 (4.3%) | 6 (0.5%) |  |  | 7 (0.5%) | 6 (0.5%) |  |
| Antithrombotic agents, N(%) | | | 0.415 |  |  |  | 0 |
| No | 3864 (85.7%) | 1293 (97.1%) | |  | 1293 (97.1%) | 1293 (97.1%) | |
| Yes | 647 (14.3%) | 39 (2.9%) |  |  | 39 (2.9%) | 39 (2.9%) |  |
| Nervous system medication, N(%) | | | 0.372 |  |  |  | 0.012 |
| No | 4066 (90.1%) | 1313 (98.6%) | |  | 1311 (98.4%) | 1313 (98.6%) | |
| Yes | 445 (9.9%) | 19 (1.4%) |  |  | 21 (1.6%) | 19 (1.4%) |  |

**Supplementary Table S2. Characteristics of included participants all-cause hospitalization (before and after PSM)**

|  | Pre-PS matching | | |  | Post-PS matching | | |
| --- | --- | --- | --- | --- | --- | --- | --- |
| Characteristics | Non-user group (n=4,511) | User group (n=1,332) | SMD |  | Non-user group (n=1,332) | User group (n=1,332) | SMD |
| Sex, N(%) |  |  | 0.07 |  |  |  | 0.028 |
| Male | 2691 (59.7%) | 840 (63.1%) | |  | 858 (64.4%) | 840 (63.1%) | |
| FeMale | 1820 (40.3%) | 492 (36.9%) | |  | 474 (35.6%) | 492 (36.9%) | |
| Residence, N(%) | |  | 0.423 |  |  |  | 0.406 |
| Urban | 2566 (56.9%) | 483 (36.3%) | |  | 747 (56.1%) | 483 (36.3%) | |
| Rural | 1945 (43.1%) | 849 (63.7%) | |  | 585 (43.9%) | 849 (63.7%) | |
| Health insurance, N(%) | | | 0.305 |  |  |  | 0.355 |
| None | 705 (15.6%) | 109 (8.2%) | |  | 234 (17.6%) | 109 (8.2%) | |
| Others | 217 (4.8%) | 22 (1.7%) |  |  | 66 (5.0%) | 22 (1.7%) |  |
| Private | 1 (0.0%) | 1 (0.1%) |  |  | 0 (0%) | 1 (0.1%) |  |
| Public | 3588 (79.5%) | 1200 (90.1%) | |  | 1032 (77.5%) | 1200 (90.1%) | |
| Age at index date, N(%) | | | 0.277 |  |  |  | 0.024 |
| 18-60 years | 1305 (28.9%) | 560 (42.0%) | |  | 576 (43.2%) | 560 (42.0%) | |
| ≥60 years | 3206 (71.1%) | 772 (58.0%) | |  | 756 (56.8%) | 772 (58.0%) | |
| BMI, N(%) | |  | 0.083 |  |  |  | 0.034 |
| ＜18.5 | 262 (5.8%) | 63 (4.7%) |  |  | 69 (5.2%) | 63 (4.7%) |  |
| 18.5-28 | 3597 (79.7%) | 1105 (83.0%) | |  | 1088 (81.7%) | 1105 (83.0%) | |
| ≥28 | 652 (14.5%) | 164 (12.3%) | |  | 175 (13.1%) | 164 (12.3%) | |
| Smoking status, N(%) | |  | 0.215 |  |  |  | 0.011 |
| No | 3552 (78.7%) | 924 (69.4%) | |  | 931 (69.9%) | 924 (69.4%) | |
| Yes | 959 (21.3%) | 408 (30.6%) | |  | 401 (30.1%) | 408 (30.6%) | |
| Alcohol status, N(%) | |  | 0.143 |  |  |  | 0.09 |
| No | 3948 (87.5%) | 1098 (82.4%) | |  | 1142 (85.7%) | 1098 (82.4%) | |
| Yes | 563 (12.5%) | 234 (17.6%) | |  | 190 (14.3%) | 234 (17.6%) | |
| FT3, pmol/L | |  | 0.082 |  |  |  | 0.026 |
| Median (Q1,Q3) | 4.30 (3.80,4.77) | 4.40 (3.90,4.85) | |  | 4.40 (3.87,4.80) | 4.40 (3.90,4.85) | |
| FT4, pmol/L | |  | 0.396 |  |  |  | 0.177 |
| Median (Q1,Q3) | 12.6 (10.4,15.1) | 10.8 (8.30,13.7) | |  | 11.4 (9.62,13.5) | 10.8 (8.30,13.7) | |
| TSH, mU/L | |  | 0.004 |  |  |  | 0 |
| Median (Q1,Q3) | 5.91 (5.14,7.77) | 7.65 (5.42,16.2) | |  | 5.92 (5.11,7.78) | 7.65 (5.42,16.2) | |
| Hypertension, N(%) | |  | 0.224 |  |  |  | 0.018 |
| No | 2189 (48.5%) | 794 (59.6%) | |  | 806 (60.5%) | 794 (59.6%) | |
| Yes | 2322 (51.5%) | 538 (40.4%) | |  | 526 (39.5%) | 538 (40.4%) | |
| Hyperlipidemia, N(%) | |  | 0.112 |  |  |  | 0.093 |
| No | 4093 (90.7%) | 1162 (87.2%) | |  | 1201 (90.2%) | 1162 (87.2%) | |
| Yes | 418 (9.3%) | 170 (12.8%) | |  | 131 (9.8%) | 170 (12.8%) | |
| Diabetes mellitus, N(%) | | | 0.213 |  |  |  | 0.017 |
| No | 4149 (92.0%) | 1290 (96.8%) | |  | 1286 (96.5%) | 1290 (96.8%) | |
| Yes | 362 (8.0%) | 42 (3.2%) |  |  | 46 (3.5%) | 42 (3.2%) |  |
| Cardiovascular system medication, N(%) | | | 1.283 |  |  |  | 0.005 |
| No | 2288 (50.7%) | 1304 (97.9%) | |  | 1305 (98.0%) | 1304 (97.9%) | |
| Yes | 2223 (49.3%) | 28 (2.1%) |  |  | 27 (2.0%) | 28 (2.1%) |  |
| Anti-diabetic medication, N(%) | | | 0.256 |  |  |  | 0 |
| No | 4316 (95.7%) | 1326 (99.5%) | |  | 1326 (99.5%) | 1326 (99.5%) | |
| Yes | 195 (4.3%) | 6 (0.5%) |  |  | 6 (0.5%) | 6 (0.5%) |  |
| Antithrombotic agents, N(%) | | | 0.415 |  |  |  | 0.023 |
| No | 3864 (85.7%) | 1293 (97.1%) | |  | 1298 (97.4%) | 1293 (97.1%) | |
| Yes | 647 (14.3%) | 39 (2.9%) |  |  | 34 (2.6%) | 39 (2.9%) |  |
| Nervous system medication, N(%) | | | 0.372 |  |  |  | 0.02 |
| No | 4066 (90.1%) | 1313 (98.6%) | |  | 1316 (98.8%) | 1313 (98.6%) | |
| Yes | 445 (9.9%) | 19 (1.4%) |  |  | 16 (1.2%) | 19 (1.4%) |  |

**Supplementary Table S3. Characteristics of included participants CVD-related hospitalization (before and after PSM)**

|  | Pre-PS matching | | |  | Post-PS matching |  |  |
| --- | --- | --- | --- | --- | --- | --- | --- |
| Characteristics | Non-user group (n=4,511) | User group (n=1,332) | SMD |  | Non-user group (n=1,332) | User group (n=1,332) | SMD |
| Sex, N(%) |  |  | 0.064 |  |  |  | 0.017 |
| Male | 2697 (59.8%) | 838 (62.9%) |  |  | 849 (63.7%) | 838 (62.9%) |  |
| FeMale | 1814 (40.2%) | 494 (37.1%) |  |  | 483 (36.3%) | 494 (37.1%) |  |
| Residence, N(%) |  |  | 0.426 |  |  |  | 0.42 |
| Urban | 2597 (57.6%) | 490 (36.8%) |  |  | 763 (57.3%) | 490 (36.8%) |  |
| Rural | 1914 (42.4%) | 842 (63.2%) |  |  | 569 (42.7%) | 842 (63.2%) |  |
| Health insurance, N(%) |  |  | 0.284 |  |  |  | 0.314 |
| None | 710 (15.7%) | 113 (8.5%) |  |  | 231 (17.3%) | 113 (8.5%) |  |
| Others | 194 (4.3%) | 22 (1.7%) |  |  | 53 (4.0%) | 22 (1.7%) |  |
| Private | 1 (0.0%) | 1 (0.1%) |  |  | 0 (0%) | 1 (0.1%) |  |
| Public | 3606 (79.9%) | 1196 (89.8%) |  |  | 1048 (78.7%) | 1196 (89.8%) |  |
| Age at index date, N(%) |  |  | 0.277 |  |  |  | 0.006 |
| 18-60 years | 1305 (28.9%) | 560 (42.0%) |  |  | 556 (41.7%) | 560 (42.0%) |  |
| ≥60 years | 3206 (71.1%) | 772 (58.0%) |  |  | 776 (58.3%) | 772 (58.0%) |  |
| BMI, N(%) |  |  | 0.064 |  |  |  | 0.022 |
| ＜18.5 | 265 (5.9%) | 65 (4.9%) |  |  | 71 (5.3%) | 65 (4.9%) |  |
| 18.5-28 | 3729 (82.7%) | 1132 (85.0%) |  |  | 1123 (84.3%) | 1132 (85.0%) |  |
| ≥28 | 517 (11.5%) | 135 (10.1%) |  |  | 138 (10.4%) | 135 (10.1%) |  |
| Smoking status, N(%) |  |  | 0.218 |  |  |  | 0.018 |
| No | 3527 (78.2%) | 914 (68.6%) |  |  | 925 (69.4%) | 914 (68.6%) |  |
| Yes | 984 (21.8%) | 418 (31.4%) |  |  | 407 (30.6%) | 418 (31.4%) |  |
| Alcohol status, N(%) |  |  | 0.142 |  |  |  | 0.078 |
| No | 3902 (86.5%) | 1083 (81.3%) |  |  | 1122 (84.2%) | 1083 (81.3%) |  |
| Yes | 609 (13.5%) | 249 (18.7%) |  |  | 210 (15.8%) | 249 (18.7%) |  |
| FT3, pmol/L |  |  | 0.11 |  |  |  | 0.047 |
| Median (Q1,Q3) | 4.30 (3.80,4.77) | 4.40 (3.92,4.82) |  |  | 4.38 (3.85,4.80) | 4.40 (3.92,4.82) |  |
| FT4, pmol/L |  |  | 0.476 |  |  |  | 0.219 |
| Median (Q1,Q3) | 12.6 (10.4,15.2) | 10.8 (8.00,13.5) |  |  | 11.2 (9.49,13.3) | 10.8 (8.00,13.5) |  |
| TSH, mU/L |  |  | 0.012 |  |  |  | 0.013 |
| Median (Q1,Q3) | 5.92 (5.15,7.78) | 7.00 (5.33,15.5) |  |  | 5.91 (5.16,7.93) | 7.00 (5.33,15.5) |  |
| Hypertension, N(%) |  |  | 0.224 |  |  |  | 0.057 |
| No | 2189 (48.5%) | 794 (59.6%) |  |  | 831 (62.4%) | 794 (59.6%) |  |
| Yes | 2322 (51.5%) | 538 (40.4%) |  |  | 501 (37.6%) | 538 (40.4%) |  |
| Hyperlipidemia, N(%) |  |  | 0.112 |  |  |  | 0.116 |
| No | 4093 (90.7%) | 1162 (87.2%) |  |  | 1210 (90.8%) | 1162 (87.2%) |  |
| Yes | 418 (9.3%) | 170 (12.8%) |  |  | 122 (9.2%) | 170 (12.8%) |  |
| Diabetes mellitus, N(%) |  |  | 0.213 |  |  |  | 0.004 |
| No | 4149 (92.0%) | 1290 (96.8%) |  |  | 1289 (96.8%) | 1290 (96.8%) |  |
| Yes | 362 (8.0%) | 42 (3.2%) |  |  | 43 (3.2%) | 42 (3.2%) |  |
| Cardiovascular system medication, N(%) |  |  | 1.283 |  |  |  | 0 |
| No | 2288 (50.7%) | 1304 (97.9%) |  |  | 1304 (97.9%) | 1304 (97.9%) |  |
| Yes | 2223 (49.3%) | 28 (2.1%) |  |  | 28 (2.1%) | 28 (2.1%) |  |
| Anti-diabetic medication, N(%) |  |  | 0.256 |  |  |  | 0.012 |
| No | 4316 (95.7%) | 1326 (99.5%) |  |  | 1327 (99.6%) | 1326 (99.5%) |  |
| Yes | 195 (4.3%) | 6 (0.5%) |  |  | 5 (0.4%) | 6 (0.5%) |  |
| Antithrombotic agents, N(%) |  |  | 0.415 |  |  |  | 0.013 |
| No | 3864 (85.7%) | 1293 (97.1%) |  |  | 1290 (96.8%) | 1293 (97.1%) |  |
| Yes | 647 (14.3%) | 39 (2.9%) |  |  | 42 (3.2%) | 39 (2.9%) |  |
| Nervous system medication, N(%) |  |  | 0.372 |  |  |  | 0 |
| No | 4066 (90.1%) | 1313 (98.6%) |  |  | 1313 (98.6%) | 1313 (98.6%) |  |
| Yes | 445 (9.9%) | 19 (1.4%) |  |  | 19 (1.4%) | 19 (1.4%) |  |

## Supplementary Table S4. Subgroup analysis of association between treatment and primary and secondary outcomes

|  | Outcomes [ HR (95%CI)] * | | | | | | | |  | |
| --- | --- | --- | --- | --- | --- | --- | --- | --- | --- | --- |
|  | 3P-MACE | P for interaction | All-cause death | P for interaction | All-cause hospitalization | P for interaction | Cardiovascular related hospitalization | P for interaction | |  |
| Age at index date |  | 0.11 |  | <0.01 |  | <0.01 |  | 0.02 | |  |
| <75 years | 0.82 (0.65, 1.04) |  | 0.14 (0.06, 0.31) |  | 0.27 (0.24, 0.31) |  | 0.79 (0.65, 0.96) |  | |  |
| ≥ 75 years | 0.64 (0.43, 0.95) |  | 0.72 (0.42, 1.22) |  | 0.11 (0.08, 0.15) |  | 0.54 (0.37, 0.79) |  | |  |
| Gender |  | 0.34 |  | <0.01 |  | <0.01 |  | 0.39 | |  |
| Male | 0.58 (0.42, 0.81) |  | 0.31 (0.17, 0.56) |  | 0.21 (0.17, 0.25) |  | 0.52 (0.40, 0.67) |  | |  |
| Female | 0.86 (0.66, 1.11) |  | 0.37 (0.20, 0.68) |  | 0.26 (0.23, 0.30) |  | 0.91 (0.72, 1.14) |  | |  |
| Smoking status |  | 0.01 |  | <0.01 |  | <0.01 |  | <0.01 | |  |
| No | 0.74 (0.58, 0.93) |  | 0.27 (0.16, 0.44) |  | 0.23 (0.20, 0.26) |  | 0.71 (0.59, 0.87) |  | |  |
| Yes | 0.81 (0.53, 1.22) |  | 0.53 (0.24, 1.19) |  | 0.30 (0.25, 0.38) |  | 0.75 (0.52, 1.09) |  | |  |
| Alcohol status |  | 0.02 |  | <0.01 |  | <0.01 |  | <0.01 | |  |
| No | 0.78 (0.62, 0.96) |  | 0.30 (0.19, 0.47) |  | 0.24 (0.22, 0.28) |  | 0.74 (0.61, 0.88) |  | |  |
| Yes | 0.63 (0.37, 1.09) |  | 0.56 (0.19, 1.67) |  | 0.25 (0.19, 0.33) |  | 0.56 (0.34, 0.92) |  | |  |
| Type of hypothyroidism |  | 0.11 |  | 0.43 |  | 0.01 |  | 0.27 | |  |
| Overt hypothyroidism | 0.92 (0.68, 1.25) |  | 0.28 (0.15, 0.50) |  | 0.29 (0.24, 0.34) |  | 0.72 (0.55, 0.94) |  | |  |
| Subclinical hypothyroidism | 0.51 (0.30, 0.87) |  | 0.27 (0.10, 0.75) |  | 0.26 (0.20, 0.34) |  | 0.96 (0.68, 1.35) |  | |  |

* If the SMD between some variables remained above 0.1 after PS matching, they were further adjusted in the Cox models.


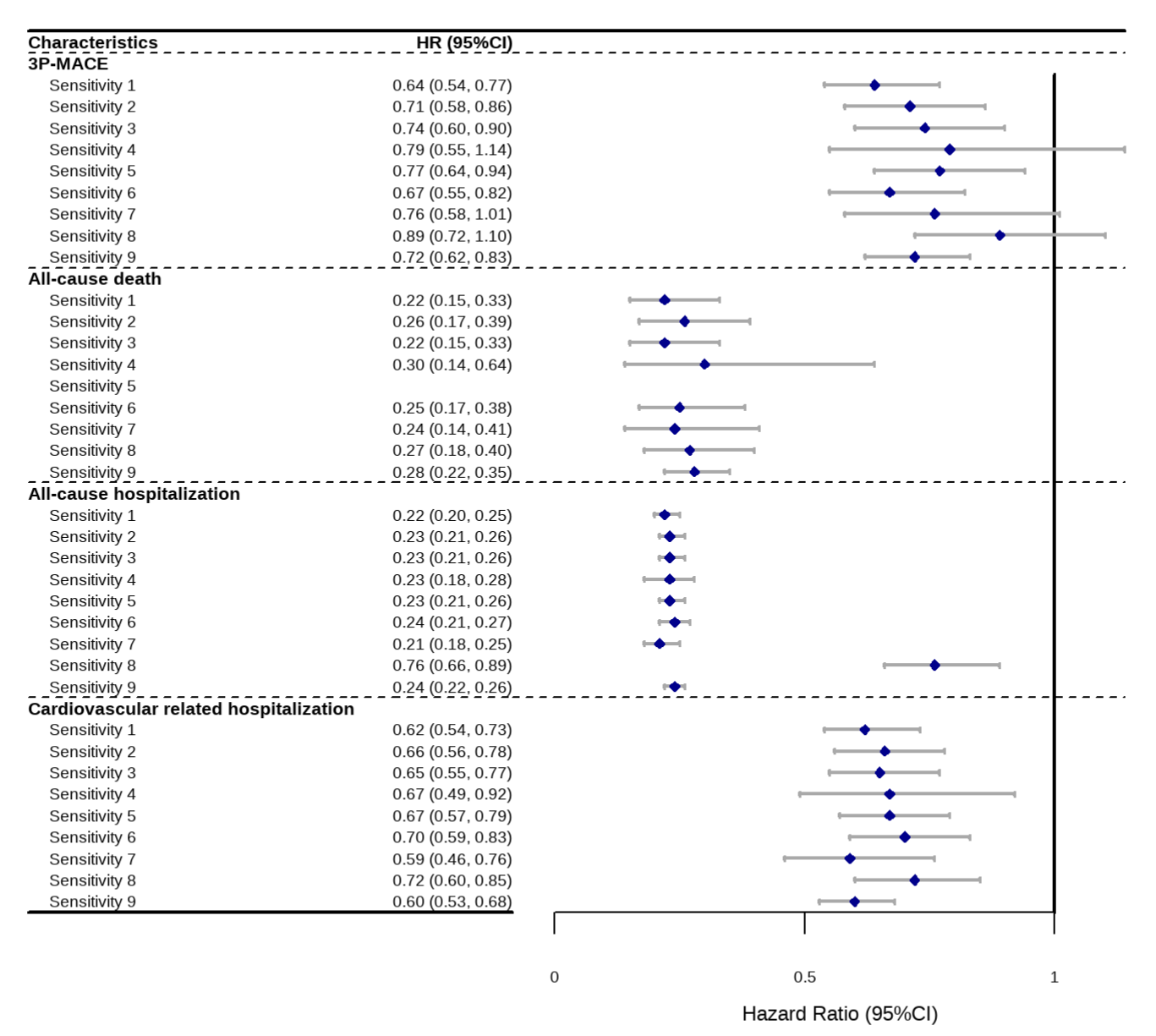


**Supplementary Figure S2. Sensitivity analysis of association between treatment and primary and secondary outcomes**

### Supplementary Table S5. Diagnosis code of diseases

| Disease | Classification | ICD-10 |
| --- | --- | --- |
| Cardiovascular disease | ischaemic heart diseases | I20-I25 |
|  | cardiomyopathy | I42 |
|  | cardiomyopathy in diseases classified elsewhere | I43 |
|  | atrioventricular and left bundle-branch block | I44 |
|  | other conduction disorders | I45 |
|  | cardiac arrest | I46 |
|  | paroxysmal tachycardia | I47 |
|  | atrial fibrillation and flutter | I48 |
|  | other cardiac arrhythmias | I49 |
|  | heart failure | I50 |
|  | complications and ill-defined descriptions of heart disease | I51 |
|  | subarachnoid haemorrhage | I60 |
|  | intracerebral haemorrhage | I61 |
|  | other nontraumatic intracranial haemorrhage | I62 |
|  | cerebral infarction | I63 |
|  | stroke, not specified as haemorrhage or infarction | I64 |
| Comorbidities for calculating CCI | myocardial infarction | I21.x, I22.x, I25.2 |
|  | congestive heart failure | I11.0, I13.0, I13.2, I25.5, I42.0, I42.5, I42.6, I42.7, I42.8, I42.9, I43.x, I50.x  P29.0 |
|  | peripheral vascular disease | I70.x, I71.x, I73.1, I73.8, I73.9, I77.1, I79.0, I79.1, I79.8, K55.1, K55.8, K55.9, Z95.8, Z95.9 |
|  | cerebrovascular disease | G45.x, G46.x, H34.0x, H34.1x, H34.2x, I60.x, I61.x, I62.x, I63.x, I64.x, I65.x, I66.x, I67.x, I68.x |
|  | dementia | F01.x, F02.x, F03.x, F04, F05, F06.1, F06.8, G13.2, G13.8, G30.x, G31.0x, G31.1, G31.2, G91.4, G94, R41.81, R54 |
|  | chronic pulmonary disease | J40.x, J41.x, J42.x, J43.x, J44.x, J45.x, J46.x, J47.x, J60.x, J61.x, J62.x, J63.x, J64.x, J65.x, J66.x, J67.x, J68.4, J70.1, J70.3 |
|  | rheumatic disease | M05.x, M06.x, M31.5, M32.x, M33.x, M34.x, M35.1, M35.3, M36.0 |
|  | peptic ulcer disease | K25.x, K26.x, K27.x, K28.x |
|  | mild liver disease | B18.x, K70.0, K70.1, K70.2, K70.3, K70.9, K71.3, K71.4, K71.5, K71.7, K73.x, K74.x, K76.0, K76.2, K76.3, K76.4, K76.8 |
|  | diabetes without chronic complication | E08, E09, E10, E11, E13, and conform to E**.0x, E**.1x, E**.6x, E**.8x, E**.9x |
|  | mild or moderate renal disease | I12.9, I13.0, I13.10, N03.x, N05.x, N18.1, N18.2, N18.3, N18.4, N18.9, Z94.0 |
|  | diabetes with chronic complications | E08, E09, E10, E11, E13, and conform to E**.2, E**.3, E**.4, E**.5 |
|  | hemiplegia or paraplegia | G04.1, G11.4, G80.0, G80.1, G80.2, G81.x, G82.x, G83.x |
|  | malignancy | C0x.x, C1x.x, C2x.x, C30.x, C31.x, C32.x, C33.x, C34.x, C37.x, C38.x, C39.x, C40.x, C41.x, C43.x, C45.x, C46.x, C47.x, C48.x, C49.x, C50, C51-58.x, C60-63.x, C76.x, C80.1, C81.x, C82.x, C83.x, C84.x, C85.x, C88.x, C9x.x |
|  | moderate or severe liver disease | I85.0x, I86.4, K70.4x, K71.1x, K72.1x, K72.9x, K76.5, K76.6, K76.7 |
|  | severe renal disease | I12.0, I13.11, I13.2, N18.5, N18.6, N19.x, N25.0, Z49.x, Z99.2 |
|  | HIV infection | B20.x |
|  | metastatic solid tumor | C77.x, C78.x, C79.x, C80.0, C80.2 |
|  | AIDS | B37.x, C53.x, B38.x, B45.x, A07.2, B25.x, G93.4x, B00, B39.x, A07.3, C46.x, C81-C96, A31.x, A15-A19, B59, Z87.01, A81.2, A02.1, B58.x, R64 |

### Supplementary Table S6. Drug code lists

| Medication for different systems | Classification | Generic and trade names |
| --- | --- | --- |
| Cardiovascular system medication | Angiotensin-converting enzyme inhibitors, oral only | Captopril, Enalapril, Benazepril, Lisinopril, Ramipril, Fosinopril, Cilazapril, Perindopril, Acertil, Imidapril, Lotensin, Kaifute, Lotensin, Eliya, Eliya, Elixir, Elinarin, Prolene, Yuening, BiPro, Monopril, Elixir, Dingtan, Captopril |
|  | Angiotensin II receptor blockers, oral only | Losartan, Valsartan, Irbesartan, Telmisartan, Candesartan, Olmesartan, Aliskiren, Suiyue, Valcke, Yifang, Pingxin, Dale, Jiafei, Mixing, Viertan, Kesuya, Beiyi, Keshilan, Daiwen, Kesu, Anbovi, Boligao, Vierya, Bilos, Aotan, Xinlitan |
|  | Beta-blockers with cardiovascular indication, oral only | Bisoprolol, Metoprolol, Atenolol, Propranolol, Betaxolol, Kangxin, Sulailuo, Betaloc, Xindean, Nadolol, Acetbutolol, Sotalol, Apulol, Esmolol |
|  | Calcium Channel Blockers, oral only | Verapamil, Diltiazem |
|  | Diuretics – low-ceiling diuretics: thiazides, oral only | Hydrochlorothiazide, Indapamide |
|  | Diuretics – low-ceiling diuretics: all except thiazides, oral only | Chlorthalidone, Indapamide, Torasemide, Furosemide, Antazoline, Lisinopril, Eplerenone, Torasemide, Valsartan, Nifedipine, Amlodipine, Guanfacine, Hydralazine, Pindolol, Atenolol, Cilnidipine, Milrinone, Amiloride, Amrinone |
|  | Diuretics – sulfonamides, oral only | Furosemide, Torasemide |
|  | Nitrates, oral or transdermal | Nitroprusside, Isosorbide Dinitrate, Nitroglycerin |
|  | Digoxin, oral only | Digoxin |
|  | Lipid modifying agents | Vastatin, Atorvastatin, Fluvastatin, Rosuvastatin, Lovastatin, Simvastatin, Pravastatin, Colesevelam, Colestipol, Cholestin, Cholestyramine, Ezetimibe, Mipomersen, PCSK9 Inhibitors, Alirocumab, Bempedoic Acid, Fenofibrate, Bezafibrate, Gemfibrozil |
| Antithrombotic agents | Heparin and other low molecular weight heparins | Heparin, Low Molecular Weight Heparin, Enoxaparin, Low Molecular Weight Heparin |
|  | Platelet aggregation inhibitors (except heparin), oral only | Sulodexide, Aspirin, Acetylsalicylic Acid, Clopidogrel, Ozagrel, Picotamide, Iloprost, Dipyridamole, Pentoxifylline, Cilostazol, Ticlopidine, Clopidogrel, Ticagrelor, Abciximab, Lamifiban, Tirofiban, Zamirlofiban, Vorapaxar, Xilariban |
|  | Anticoagulants (vitamin K, direct thrombin inhibitors, direct factor Xa inhibitors), oral only | Hirudin, Argatroban, Coumarin, Warfarin, Acenocoumarol, Dabigatran Etexilate, Rivaroxaban, Apixaban, Edoxaban |
| Antihyperglycemic medication | metformin | Metformin, Diabinese, Medicone, Glucophage, Precose, Amaryl, Glucotrol, Liraglutide, Januvia, Actos, Starlix, Byetta, Xiaoke, Insulin |
|  | thiazolidinedione | Pioglitazone, Actoplus Met, Kazano, Oseni, Invokana, Canagliflozin, Jardiance, Farxiga, Steglatro, Glyxambi, Synjardy, Qtern, Oseni, Rybelsus, Rosiglitazone, Glimepiride, Glipizide, Glucovance, Tolinase, Gliclazide, Sulfonylureas, Repaglinide, Nateglinide, DPP-4 inhibitors, SGLT2 inhibitors |
|  | sodium-glucose cotransporter 2 Inhibitor | Canagliflozin, Empagliflozin, Dapagliflozin, Alogliptin, Ertugliflozin, Sitagliptin, Vildagliptin, Luseogliflozin, Tofogliflozin, Teneligliptin |
|  | Glucagon-like peptide-1 receptor agonist | Exenatide [Byetta], Liraglutide [Victoza], Liraglutide [Saxenda], Semaglutide [Ozempic], Exenatide Extended-Release [Bydureon], Somaglutide, Abiglutide, Dulaglutide [Trulicity], Semaglutide [Rybelsus], Lixisenatide [Lyxumia] |
| Nervous system medication | Antidepressants - non-selective monoamine reuptake inhibitors, oral only | Imipramine, Clomipramine, Amitriptyline, Dosulepin |
|  | Antidepressant - selective serotonin or selective serotonin/norepinephrine reuptake inhibitors, oral only | Desipramine, Maprotiline, Nortriptyline, Protriptyline, Amoxapine, Fluoxetine, Paroxetine, Sertraline, Citalopram, Venlafaxine, Duloxetine, Doxepin, Mianserin, Bupropion, Reboxetine, Trazodone |
|  | Antipsychotics | Chlorpromazine, Perphenazine, Trifluoperazine, Clotiapine, Thioridazine, Flupenthixol, Fluphenazine, Pimozide, Flupenthixol, Pimozide, Sulpiride, Clozapine, Risperidone |
|  | Lithium, oral only | Lithium Carbonate |
|  | Benzodiazepines, oral only | Diazepam, Oxazepam, Lorazepam, Alprazolam, Midazolam, Clonazepam, Nitrazepam, Clonazepam, Clorazepate, Flurazepam, Triazolam |
|  | Hypnotics (non-benzodiazepines), oral only | Barbital, Pentobarbital, Secobarbital, Hexobarbital, Thiopental, Luminal, Amobarbital, Temazepam, Chloral Hydrate, Zolpidem, Zopiclone, Zaleplon |
| Thyroid medication | Levothyroxine | Synthroid, Levothyroxine, Letrox, Methimazole |
|  | Other thyroid hormones | Liothyronine, Tiratricol, Thyroid Tablets |
|  | Antithyroid agent | Methimazole, Propylthiouracil, Tapazole, Thiouracil, Carbimazole, Sodium Perchlorate, Potassium Perchlorate, Dibromotyrosine, Diiodotyrosine |
